# PSMB9 Orchestrates Tumor Immune Landscape and Serves as a Potent Biomarker for Prognosis and T Cell-Based Immunotherapy Response

**DOI:** 10.3390/cimb47090712

**Published:** 2025-09-01

**Authors:** Xinran Ma, Qi Zhu, Zhiqiang Wu, Weidong Han

**Affiliations:** 1 School of Medicine, Nankai University, Tianjin 300071, China; hnjzmxr@hotmail.com; 2 Department of Bio-Therapeutic, The First Medical Center, Chinese PLA General Hospital, Beijing 100853, China; qizhu9951@gmail.com (Q.Z.); wuzhiqiang1006@163.com (Z.W.)

**Keywords:** PSMB9, immune process, T cell-based immunotherapy, immunotherapy response, prognostic biomarker

## Abstract

Proteasome subunit beta type-9 (PSMB9), a member of the proteasome beta subunit family, encodes the pivotal β1i component of the immunoproteasome. PSMB9 plays a crucial role in antigen processing and presentation; however, its comprehensive role in orchestrating a tumor-immune landscape and regulating the anti-tumor immune responses remains unexplored. Here we investigated the context-dependent functions of PSMB9 by integrating multi-omics data from The Cancer Genome Atlas, Genotype-Tissue Expression database, Human Protein Atlas, Tumor Immunotherapy Gene Expression Resource, and multiple other databases. Moreover, we explored the predictive value of PSMB9 in multiple immunotherapy cohorts and investigated its functional relevance in CAR-T therapy using genome-scale CRISPR/Cas9 screening, gene knockout cell line in vitro, and clinical cohort validation. We found widespread dysregulation in PSMB9 across cancers, predominantly upregulated in most malignancies and associated with advanced pathological stages in specific contexts. PSMB9 was also broadly and negatively correlated with tumor stemness indices. Crucially, PSMB9 expression was robustly linked to anti-tumor immunity by being significantly correlated with immune-pathway activation (e.g., IFN response, cytokine signaling), immune regulatory and immune checkpoint gene expression, and enhanced infiltration of T cells across nearly all tumor types. Consequently, elevated PSMB9 predicted superior response to immune checkpoint inhibitors in multiple cohorts, showing comparable predictive power to established predictive signatures. Furthermore, CRISPR/Cas9 screening identified PSMB9 loss as a novel mechanism of resistance to CD19 CAR T cell therapy, with PSMB9-deficient tumor cells exhibiting a survival advantage under CAR-T pressure, supported by trends in clinical CAR-T outcomes. Our study uncovers PSMB9 as a previously unrecognized critical regulator of the tumor immune landscape in a pan-cancer scope, whose expression orchestrates key immune processes within the tumor microenvironment and serves as a potent biomarker for patient prognosis. Critically, we first established PSMB9 as a novel prognostic indicator for both checkpoint blockade and CAR-T cell therapies, highlighting its dual role as a crucial immune modulator and a promising biomarker for guiding T cell-based immunotherapy strategies across diverse human cancers.

## 1. Introduction

In recent years, promising modalities that harness the power of the immune system have offered new approaches to effective and personalized therapeutic strategies, revolutionizing the landscape of cancer treatment. Among numerous immunotherapy strategies, T cell-based immune checkpoint inhibitors (ICIs) and adoptive cell therapies like chimeric antigen receptor T (CAR-T) cell therapy have exhibited impressive efficacy in combating cancer [1]. Despite this success, there are still a considerable number of patients who lack responses to immunotherapy [2]. In this context, identification of reliable biomarkers that can effectively predict treatment response and patient outcomes remains a task of priority.

Proteasome subunit beta type-9 (PSMB9), also known as low-molecular-mass protein 2 (LMP2), is a catalytic subunit of the immunoproteasome that plays a crucial role in antigen processing and presentation by major histocompatibility complex (MHC) class I molecules [3]. In hematopoietic cells, PSMB9 and PSMB10 replace the standard proteasome subunits β1c, β2c, and β5c in the inner two rings of the 20S proteasome, forming the immunoproteasome, causing protein degradation, and generating the immunogenic peptide epitopes for MHC-I presentation [4,5].

Accumulating evidence has highlighted aberrant PSMB9 expression as a prognostic factor in various pathological conditions, especially in autoimmune diseases [6,7,8,9,10]. Considering the role of PSMB9 as antigen-presenting machinery, the possible role of PSMB9 in shaping the immunotherapy response of cancers has garnered increasing attention recently. A small group study found high PSMB9 levels related to durable clinical benefit after anti-PD1 treatment in non-small-cell lung cancers (NSCLCs) [11], suggesting that PSMB9 could serve as a predictive biomarker for identifying patients likely to benefit from immunotherapy. Additionally, Hu et al. have reported a tumor-suppressive role of PSMB9 in melanoma, prompting PSMB9 as a potential target for treatment strategies [12]. Nevertheless, current evidence is limited to a few cancer types with small sample sizes, with no systematic evaluation of its immune regulatory or predictive role across diverse malignancies. Given the multifaceted roles of PSMB9 in cancer biology and immunotherapy, a comprehensive analysis of its prognostic value and impact on the immunotherapy response across different cancer types is therefore necessary. Here, we established this pan-cancer analysis to systematically elucidate the role of PSMB9 in orchestrating tumor immune processes and its potential as a novel predictive biomarker for T cell-based immunotherapy responses in diverse malignancies.

In this study, we performed a comprehensive, multi-level pan-cancer analysis of PSMB9 expression, its impact on prognosis, tumor stemness, tumor immune responses, immune regulatory and immune checkpoint gene expression, immune cell infiltration, as well as its predictive value on immunotherapy outcome. Our results also indicated a previously unreported association between PSMB9 and CAR-T cell therapy response. This study aims to shed light on the clinical relevance of PSMB9 as a prognostic biomarker and predictive indicator for immunotherapy efficacy, especially on T cell-based treatment, contributing to the ongoing efforts to optimize cancer treatment strategies and improve patient outcomes in the era of precision medicine and personalized immunotherapy.

## 2. Materials and Methods

### 2.1. Expression Profiles

PSMB9 expression profiles in 35 different normal tissues were generated using the Genotype-Tissue Expression (GTEx) database (https://www.gtexportal.org/, accessed on 6 June 2025). The RNA-seq tissue data was reported as nTPM (normalized protein-coding transcripts per million), corresponding to mean values of the different individual samples from each tissue.

The expression of PSMB9 in main human tissues at the single-cell level was generated by single-cell portal of the Human Protein Atlas (HPA) (www.proteinatlas.org, accessed on 16 June 2025) [13], with preprocessing following HPA’s standardized protocols (https://www.proteinatlas.org/about/assays+annotation#normalization_rna, accessed on 16 June 2025).

Cancer tissue transcriptional data and normal human tissue data were from the TCGA Pan-cancer cohort and GTEx database, respectively. R software (version 4.3.2) was used to calculate expression differences between normal and tumor tissues for each cancer type and to perform significance analysis of the differences using the unpaired Wilcoxon Rank Sum and Signed Rank Tests. The abbreviations of all cancer types are presented in Supplemental Table S1.

Stage-specific analysis of PSMB9 was conducted utilizing the GEPIA2 (http://gepia.cancer-pku.cn, accessed on 30 June 2025) webtool [14], with data transformed using log2(x + 0.001). Differences in expression were evaluated through ANOVA complemented by Tukey’s post hoc test. To guarantee result reliability, cancer types with fewer than 3 samples per stage were removed from the analysis.

Immunofluorescence images from HPA [13] were used to show the subcellular localization of PSMB9. Representative immunohistochemistry (IHC) staining images illustrating PSMB9 expression in normal breast tissues and breast cancer tissues were also sourced from the HPA database.

### 2.2. Prognosis Analysis

Survival analysis was based on the uniformly normalized pan-cancer dataset TCGA TARGET GTEx (N = 19,131), retrieved from the UCSC Xena Browser. After excluding samples with undetectable expression levels and those with a follow-up duration of less than 30 days and cancer types with fewer than 10 samples, we employed the R package “survival” (v3.2-7) to build a Cox proportional hazards regression model to analyze associations between PSMB9 expression and prognosis across various cancers.

### 2.3. Genomic Alterations and Modification Analysis

The cBioPortal for Cancer Genomics (http://www.cbioportal.org, accessed on 10 June 2025) is an open-access database for the exploration, analysis, and visualization of multidimensional cancer genomics data [15,16]. Here, cBioPortal was used to investigate the genomic alteration types and frequencies across different cancers. Visualization of the genomic alteration rate was achieved by the cBioPortal web tool. Normalized pan-cancer dataset TCGA TARGET GTEx (N = 19,131) from the UCSC Xena database (https://xenabrowser.net/, accessed on 6 June 2025) [17] was downloaded. We extracted the expression data of PSMB9 and 60 RNA modification marker genes (m1A (10), m5C (13), m6A (21)) in each sample and analyzed the Pearson correlation between PSMB9 and the marker genes of these three RNA modification categories. The sangerbox3.0 database (http://sangerbox.com/home.html, accessed on 16 June 2025) [18] was accessed for RNA modification marker gene set acquisition.

### 2.4. Analysis of Tumor Stemness Correlations

Analysis of tumor stemness correlations was based on the normalized pan-cancer dataset TCGA TARGET GTEx (N = 19,131), retrieved from the UCSC Xena Browser. Building on the methodology of Tathiane et al. [19], we derived three cancer stemness indices (DNAss, EREG-METHss, and ENHss) from mRNA expression and methylation feature analyses. The Sangerbox3.0 database (http://sangerbox.com/home.html, accessed on 16 June 2025) [18] was accessed to compute the Pearson correlation coefficients between these stemness scores and PSMB9 expression for all tumor samples and to visualize into lollipop plots.

### 2.5. Gene Set Enrichment Analyses

The differentially expressed genes (DEGs) between low- and high-PSMB9 subgroups were stratified based on their PSMB9 mRNA expression levels. The top 30% of patients were categorized as the high-PSMB9 subgroup, while the bottom 30% comprised the low-PSMB9 subgroup. Genes exhibiting adjusted *p* values < 0.05 were identified as DEGs. Subsequent differential expression analyses were conducted utilizing the “limma” R package [20] (v3.54.0). The list of DEGs between low- and high-PSMB9 subgroups across individual cancers is provided in Supplementary Table S2. The hallmark gene set file (h.all.v7.4.symbols.gmt) encompassing 50 hallmark gene sets was obtained from the Molecular Signatures Database (MSigDB) website [21] (https://www.gsea-msigdb.org/gsea/index.jsp, accessed on 6 June 2025) and employed to calculate the normalized enrichment score (NES) and false discovery rate (FDR) for each biological process within distinct cancer types. gene set enrichment analysis was executed utilizing the R packages “clusterProfiler” (v3.10.1) and “GSVA” (v2.2.0) [22,23], with the outcomes presented in a bubble plot generated by the R package “ggplot2” (v3.5.2).

### 2.6. Immune Cell Infiltration Analysis

We downloaded the uniformly normalized pan-cancer dataset (N = 19,131) from the UCSC database (https://xenabrowser.net/, accessed on 27 June 2025). Further, we extracted the expression data of PSMB9 and 150 marker genes from five categories of immune pathways (chemokine, receptor, MHC, immunoinhibitor, and immunostimulator) in each sample. Using R software (version 4.3.2), we analyzed the Pearson correlation between PSMB9 and the marker genes of these five immune pathway categories. The Pearson correlation between PSMB9 and 60 inhibitory and stimulatory immune checkpoint pathway genes was also analyzed. We also extracted the gene expression profiles of pan-cancer data from UCSC Xena of each tumor, respectively, and mapped these profiles to GeneSymbol. Furthermore, we utilized the deconvo_xCell method from the R package IOBR (version 0.99.9) to evaluate the immune cell infiltration as well as the infiltration scores of ImmuneScore, StromaScore, and MicroenvironmentScore for each patient in each tumor based on gene expression. Ultimately, we obtained the infiltration scores of 67 types of immune cells from 10,180 tumor samples. We then used the corr.test function from the R package psych (version 2.1.6) to calculate the Pearson’s correlation coefficient between genes and immune cell infiltration scores in each tumor, aiming to identify significantly correlated immune infiltration scores.

### 2.7. Immunotherapy Analysis

Tumor Immunotherapy Gene Expression Resource (TIGER) database [24] (http://tiger.canceromics.org/, accessed on 16 June 2025) was used to investigate the connection between PSMB9 expression and ICI response. In total, 25 cohorts involving ICIs therapy were accessed to validate the predictive efficacy of PSMB9 in immunotherapy response with their information details listed in Supplemental Table S3. Patients from cohort PRJEB23709, GSE100797 and PRJEB25780 [25,26] were stratified into low-expression and high-expression groups based on median PSMB9 expression to compare respective Kaplan–Meier survival curves or calculate the proportion of responders and non-responders in high- and low-PSMB9 subgroups across different tumor types and immunotherapeutic strategies. These validation data were used without overlapping results. The predictive value of PSMB9 for immunotherapies was compared with other previously reported immune response signatures using the correlation matrix of TIGER database.

We retrieved PSMB9 expression data for each sample from the pan-cancer dataset of the UCSC Xena database. Additionally, we obtained level-4 Simple Nucleotide Variation (SNV) datasets for all TCGA samples, which had been processed using MuTect2, from the GDC portal (https://portal.gdc.cancer.gov/, accessed on 16 June 2025). Leveraging the R package maftools (v2.8.05), we computed the tumor mutation burden (TMB) and microsatellite instability (MSI) for each tumor type. We then integrated the TMB/MSI metrics with the corresponding gene expression profiles and performed Pearson correlation analyses within each cancer type.

### 2.8. CAR-T Cell Generation

The CAR-T cells utilized herein were generated according to established protocols from our prior publications [27,28,29,30]. Lentiviral vectors encoding the CAR constructs were assembled within the pRRLSIN-EF-1α-GFP backbone. To produce viral particles, 293T cells underwent cotransfection with this vector alongside the PSPAX2 and PMD2G packaging plasmids. Supernatants containing lentivirus were collected after 48 h and cryopreserved at −80 °C. Primary human T lymphocytes were isolated from healthy donor peripheral blood mononuclear cells (PBMCs). T cell activation was initiated using plates pre-coated for 12–16 h at 4 °C with anti-CD3 antibody (OKT3, Takara, Kyoto, Japan; 1 µg/mL) and RetroNectin (Takara). Activated T cells were expanded in X-VIVO 15 medium (Lonza, Basel, Switzerland) supplemented with 10% fetal bovine serum (Gibco) and recombinant human IL-2 (300 U/mL; PeproTech, Cranbury, NJ, USA). Two days post-activation, activated T cells were combined with lentiviral supernatant on RetroNectin-coated plates in the presence of polybrene (4 µg/mL; Sigma–Aldrich, St. Louis, MO, USA). This mixture underwent centrifugation (1000× *g*, 10 min). Following a 24 h incubation period, the medium was refreshed to minimize residual polybrene exposure. Subsequently, transduced CAR-T cells were maintained in X-VIVO 15 medium containing IL-2 (300 U/mL). All procedures were conducted in accordance with the Declaration of Helsinki and were approved by the Research Ethics Board of Chinese PLA General Hospital (approval number: KY-2022-8-67-1). 

### 2.9. CRISPR/Cas9 Screening

The process of CRISPR/Cas9 screening has been described in the previous work of our group [29,30]. Nalm6 cells were transfected with lentivirus containing the Brunello library, and subsequent selection of cells harboring stable lentiviral integrations was achieved through puromycin treatment. These transduced Nalm6 cells were co-cultured with CD19 CAR T cells or control T cells at a 1:50 effector-to-target (E:T) ratio. Control or CD19 CAR T cells were replenished every 3 days at the same E:T ratio during the culture period. Cell collection was facilitated by utilizing a death cell removal magnetic bead kit (Miltenyi, Bergisch Gladbach, Germany) for genomic DNA analysis on day 15 of the experiment. Subsequent to filtering out low-quality sequencing reads, the obtained reads were aligned to the single-guide RNA (sgRNA) sequences, and the read count for each sgRNA was tabulated to construct a sgRNA count table. The sgRNA data within this table underwent normalization for subsequent significance analysis. The MAGeCK v0.5.7 algorithm was employed to analyze the sgRNA read counts, enabling the identification of genes displaying significantly enriched sgRNAs as per predefined criteria of log2 fold change and *p* value.

### 2.10. Establishment of PSMB9^KO^ Cell Line

The Cas9 protein and sgRNA were electroporated into Nalm6 cells to knock out the expression of PSMB9. To prepare the ribonucleoprotein mixture, 6 μg of the Cas9 protein (Integrated Device Technology, Inc., San Jose, CA, USA) and 6 μg of sgRNA (Integrated Device Technology, Inc.) were mixed together in Opti-MEM medium (Gibco, Grand Island, CA, USA) to create a total volume of 20 μL ribonucleoprotein mixture, incubated at room temperature for 20 min and transferred to ice immediately before electroporation. Nalm6 cells (106) washed once by Opti-MEM medium (Gibco) were resuspended in 80 μL of precooled Opti-MEM medium, sitting on ice for 5 min, adding the ribonucleoprotein mixture, fully mixed and transferred into a 4 °C precooled 2 mm cuvette (Harvard Apparatus BTX, Holliston, MA, USA). Then, the cells were electroporated using a BTX Gemini System (Harvard Apparatus BTX) at 250 V for 5 ms. Following electroporation, the cells were immediately transferred into 2 mL of 37 °C prewarmed medium and cultured at 37 °C and 5% CO_2_. The gRNA targeting sequences used in this study were as follows: PSMB9 sgRNA: ACCAACCGGGGACTTACCCC; negative control gRNA: AAAUGUGAGAUCAGAGUAAU.

### 2.11. Western Blot

Tumor cells were lysed in RIPA buffer containing protease and phosphatase inhibitors (Roche, Basel, Switzerland). Equal amounts of protein were separated by MES-SDS on 10% Bis-Tris gels (Genscript, Nanjing, China) and transferred onto PVDF membranes (Millipore, Billerica, MA, USA). Membranes were blocked with 5% non-fat milk in TBST for 1 h at room temperature, followed by incubation with primary antibody overnight at 4 °C. After TBST washes, membranes were incubated with HRP-conjugated secondary antibodies for 1 h at room temperature. Protein bands were visualized using enhanced chemiluminescence (ECL) substrate (YangGuangBio, Beijing, China) and imaged with the imaging system (ServiceBio, Beijing, China). Primary antibodies include the following: PSMB9 rabbit mAb (A9549, Abclonal, Wuhan, China), β-actin mouse mAb (AC004, Abclonal).

### 2.12. Statistical Analysis

The measurement results are displayed as mean ± standard deviations. Group differences were assessed using two-way ANOVA test with multiple comparisons. Prognostic outcomes, including hazard ratios and significance levels, were examined through Cox regression models and log-rank testing. Correlations between PSMB9 and RNA modifications, immunomodulatory genes, tumor stemness, immune checkpoint genes, and immune cell infiltration were determined via Pearson’s correlation. A significance threshold of *p* < 0.05 was applied.

## 3. Results

### 3.1. PSMB9 Displays Dysregulated Expression Patterns Across Diverse Human Cancers

**Figure 1:**
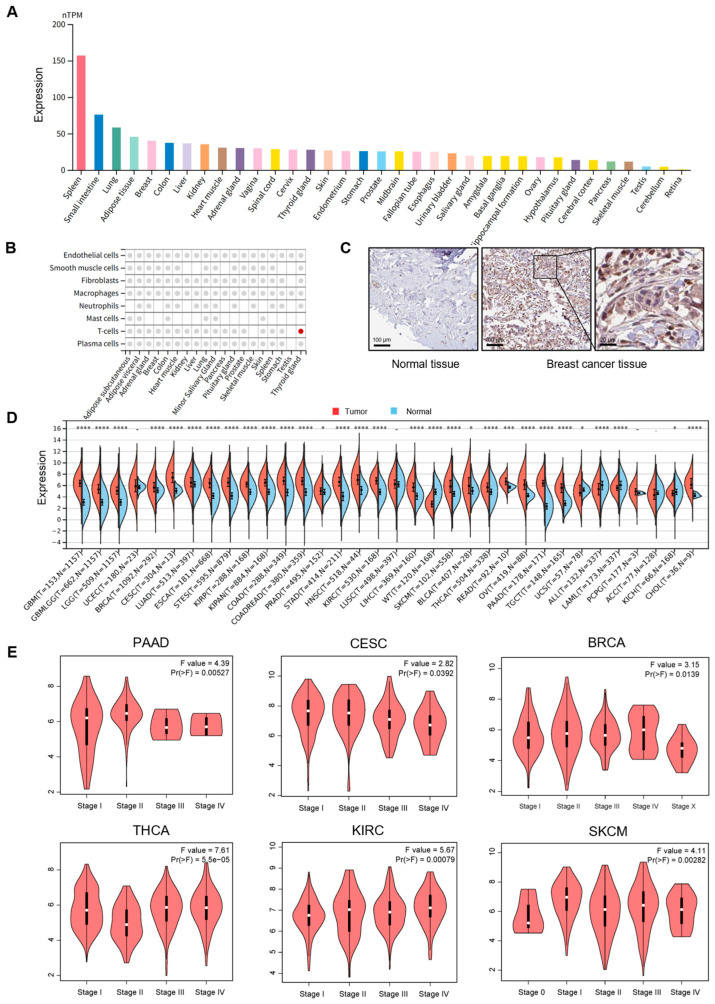
PSMB9 expression patterns across normal tissues and tumor types: (**A**) PSMB9 expression profiles in normal tissues from the GTEx dataset, with color coding stratified by tissue groups that share common functional characteristics. (**B**) PSMB9 expression in main normal tissues at single-cell levels. Colored dots denote tissues where PSMB9 exhibits core cell type specificity, while gray dots indicate the presence of PSMB9 expression in the tissue without predicted enrichment. (**C**) Representative immunohistochemistry (IHC) staining images from the HPA database (**C**) show significantly higher PSMB9 expression in breast cancer tissues than in normal breast tissues, sourced from the HPA database. (**D**) Differences in PSMB9 expression between tumor and normal tissues, analyzed using integrated data from the TCGA and GTEx databases. (**E**) Correlation analyses between PSMB9 expression and pathological stages in PAAD, CESC, BRCA, THCA, KIRC, and SKCM. * *p* < 0.05; *** *p* < 0.001; **** *p* < 0.0001.

We analyzed the GTEx database to explore the expression profile of PSMB9 in normal human tissues (Figure 1A). Notably, PSMB9 expression displayed marked variability across different tissues, with particularly high levels observed in the spleen, a major immune organ. We further examined the cell-type specificity of PSMB9 in normal tissues at the single-cell level (Figure 1B and Figure S1) and found that its expression was significantly enriched in T lymphocytes within thyroid tissue. In other tissues, although PSMB9 expression could be detected, no obvious cell-type preference was observed. To further clarify the expression level of PSMB9 in tumors, we first examined the expression level of PSMB9 in different tumor cell lines. PSMB9 expression was relatively higher in brain cancer, head and neck cancer, bile duct cancer, kidney cancer, leukemia, lymphoma, and myeloma, among which lymphoma showed the highest expression (Figure S2).

To compare PSMB9 protein levels between normal and tumor tissues, representative immunohistochemical (IHC) staining graphics were obtained from the HPA database (Figure 1C), revealing significantly higher PSMB9 expression in breast cancer tissues relative to normal breast tissues, indicative of its aberrant expression in tumors. To further characterize the subcellular localization of PSMB9, we obtained immunofluorescence images of two different cell lines from the HPA database, which showed that PSMB9 is predominantly concentrated in the cytoplasm (Figure S3).

To systematically dissect the differential expression patterns of PSMB9 between diverse tumor tissues and their matched normal counterparts, we integrated data from the TCGA and GTEx databases and analyzed PSMB9 expression in multiple cancer types and their corresponding normal tissues (Figure 1D). This analysis revealed elevated PSMB9 expression in most malignancies, including glioblastoma multiforme (GBM), glioma (GBMLGG), brain lower grade glioma (LGG), breast invasive carcinoma (BRCA), endocervical adenocarcinoma (CESC), lung adenocarcinoma (LUAD), esophageal carcinoma (ESCA), stomach and esophageal carcinoma (STES), kidney renal papillary cell carcinoma (KIRP), colon adenocarcinoma (COAD), prostate adenocarcinoma (PRAD), stomach adenocarcinoma (STAD), head and neck squamous cell carcinoma (HNSC), kidney renal clear cell carcinoma (KIRC), liver hepatocellular carcinoma (LIHC), skin cutaneous melanoma (SKCM), bladder urothelial carcinoma (BLCA), thyroid carcinoma (THCA), rectum adenocarcinoma (READ), ovarian serous cystadenocarcinoma (OV), pancreatic adenocarcinoma (PAAD), testicular germ cell tumors (TGCTs), acute myeloid leukemia (LAML), kidney chromophobe (KICH), and cholangiocarcinoma (CHOL). In contrast, reduced expression was observed in uterine carcinosarcoma (UCS), acute lymphoblastic leukemia (ALL), and high-risk Wilms tumor (WT).

We further investigated the association between PSMB9 expression and tumor stage by integrating TCGA pan-cancer data (Figure 1E), with results revealing significant stage-dependent differences in PSMB9 expression across multiple malignancies, exhibiting cancer-specific regulatory directions. Specifically, a negative correlation between PSMB9 expression and pathological stage was found in advanced-stage PAAD, CESC, and BRCA. On the contrary, we observed positive correlations between PSMB9 expression and early-stage KIRC and SKCM, while a negative correlation was noted in early-stage THCA. These findings suggest that PSMB9 expression may be closely associated with the malignant progression of specific tumor types, exhibiting distinct association patterns in different cancers.

### 3.2. PSMB9 Expression Levels Exert Distinct Prognostic Impacts Across Different Tumor Types

**Figure 2:**
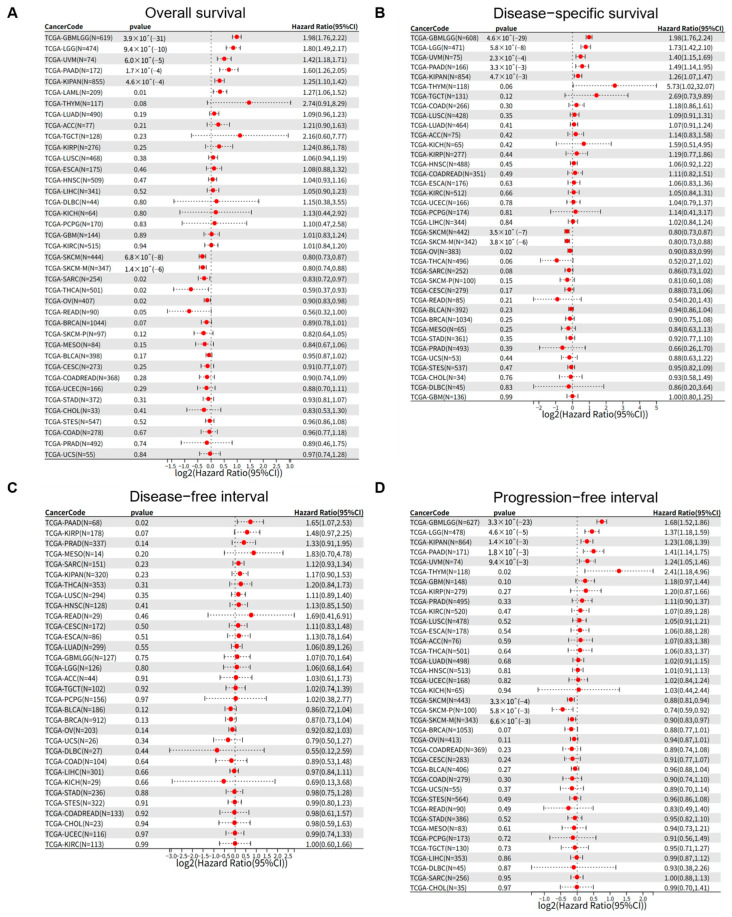
PSMB9 exerts varied clinical prognostic impacts on tumors. The associations between PSMB9 expression and (**A**) overall survival, (**B**) disease-specific survival, (**C**) disease-free interval, and (**D**) progression-free interval were explored by Cox survival analysis across different tumor types.

To systematically interrogate the prognostic relevance of PSMB9 across human malignancies, we leveraged pan-cancer survival data from the TCGA database and performed Cox proportional-hazards modeling. PSMB9 transcript abundance independently predicted overall survival (OS) in 12 tumor contexts (Figure 2A). High PSMB9 expression was associated with significantly reduced OS in GBMLGG (HR = 1.98 [95% CI, 1.76–2.22], *p* < 0.001), LGG (HR = 1.80 [95% CI, 1.49–2.17], *p* < 0.001), UVM (HR = 1.42 [95% CI, 1.18–1.71], *p* < 0.001), PAAD (HR = 1.60 [95% CI, 1.26–2.05], *p* < 0.001), KIPAN (HR = 1.25 [95% CI, 1.10–1.42], *p* < 0.001) and LAML (HR = 1.27 [95% CI, 1.06–1.52], *p* = 0.01), whereas it conferred a survival benefit in SKCM (HR = 0.80 [95% CI, 0.73–0.87], *p* < 0.001), SKCM-M (HR = 0.80 [95% CI, 0.74–0.88], *p* < 0.001), SARC (HR = 0.83 [95% CI, 0.72–0.97], *p* = 0.02), THCA (HR = 0.59 [95% CI, 0.37–0.93], *p* = 0.02), OV (HR = 0.90 [95% CI, 0.83–0.98], *p* = 0.02) and READ (HR = 0.56 [95% CI, 0.32–1.00], *p* = 0.05). Concordant patterns were observed for disease-specific survival (DSS). Elevated PSMB9 independently forecast inferior DSS in GBMLGG (HR = 1.98 [95% CI, 1.76–2.24], *p* < 0.001), LGG (HR = 1.73 [95% CI, 1.42–2.10], *p* < 0.001), UVM (HR = 1.40 [95% CI, 1.15–1.69], *p* < 0.001), PAAD (HR = 1.49 [95% CI, 1.14–1.95], *p* = 0.0033) and KIPAN (HR = 1.26 [95% CI, 1.07–1.47], *p* = 0.0047), whereas diminished PSMB9 expression predicted compromised DSS in SKCM (HR = 0.80 [95% CI, 0.73–0.87], *p* < 0.001), SKCM-M (HR = 0.80 [95% CI, 0.73–0.88], *p* < 0.001) and OV (HR = 0.90 [95% CI, 0.83–0.99], *p* = 0.02) (Figure 2B). Restricting the endpoint to disease-free interval (DFI) revealed a significant inverse association solely in PAAD (HR = 1.65 [95% CI, 1.07–2.53], *p* = 0.02) (Figure 2C). In analyses of progression-free interval (PFI), upregulated PSMB9 remained a robust negative determinant in GBMLGG (HR = 1.68 [95% CI, 1.52–1.86], *p* < 0.001), LGG (HR = 1.37 [95% CI, 1.18–1.59], *p* < 0.001), KIPAN (HR = 1.23 [95% CI, 1.08–1.39], *p* = 0.0014), PAAD (HR = 1.41 [95% CI, 1.14–1.75], *p* = 0.0018), UVM (HR = 1.24 [95% CI, 1.05–1.46], *p* = 0.0094), and THYM (HR = 2.41 [95% CI, 1.18–4.96], *p* = 0.02) yet correlated with prolonged PFI in SKCM (HR = 0.88 [95% CI, 0.81–0.94], *p* < 0.001), SKCM-P (HR = 0.74 [95% CI, 0.59–0.92], *p* = 0.0058), and SKCM-M (HR = 0.90 [95% CI, 0.83–0.97], *p* = 0.0066) (Figure 2D). Collectively, these findings establish PSMB9 as a context-dependent biomarker whose expression delineates divergent clinical trajectories across multiple cancer lineages.

### 3.3. PSMB9 Alteration Profiles and Their Role in Tumor Stemness

**Figure 3:**
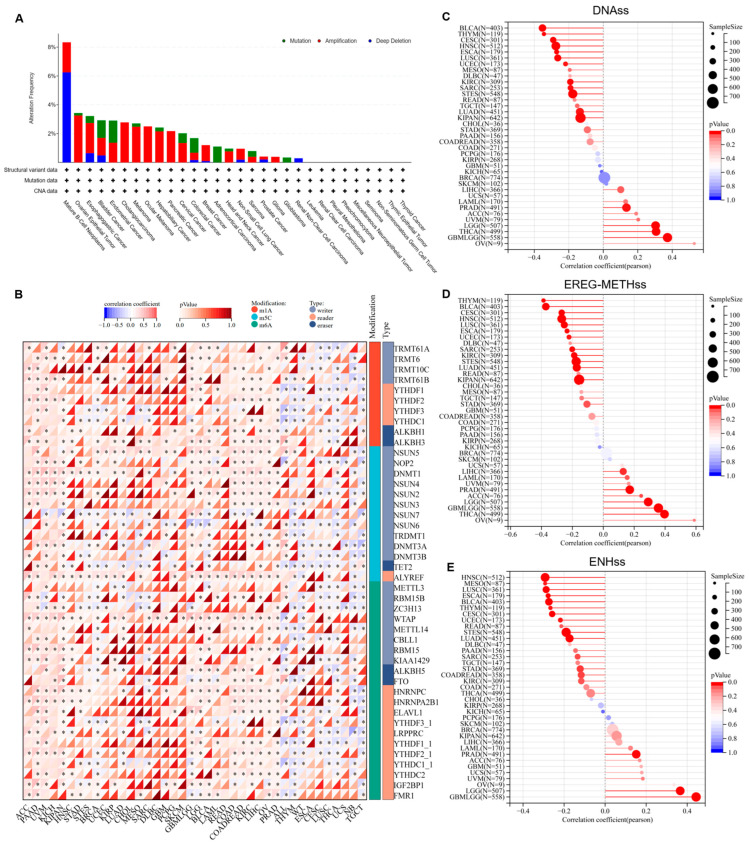
PSMB9 alteration profiles and their expression correlations with RNA modification genes and tumor stemness indicators: (**A**) PSMB9 alteration (mutation, amplification, and deep deletion) in different tumor types. (**B**) Pearson correlation analysis of PSMB9 expression and 44 class III RNA modification genes. (**C**–**E**) Pearson correlation analysis of PSMB9 expression and tumor stemness indicators including (**C**) DNAss, (**D**) EREG-METHss, and (**E**) ENHss. DNAss, DNA expression-based; EREG.EXPss, epigenetically regulated RNA expression-based; and ENHss, enhancer elements/DNA methylation-based. The size of the circles corresponds to sample sizes. The color gradient of the bars indicates the significance of the *p* value, with the red color corresponding to more significant *p* values. * *p* < 0.05.

In order to further elucidate the characteristics of PSMB9 and its role in tumorigenesis and progression, we analyzed the alteration profiles of PSMB9, its RNA modification features, and its potential regulatory role in tumor stemness. Mutation spectrum analysis based on the TCGA pan-cancer dataset revealed that the proportion of PSMB9 alterations was highest in mature B-cell neoplasms, approximately 8%, with deep deletion being the predominant type, accounting for about 3/4 of all alterations (Figure 3A). In most other tumor types, the alteration rate of PSMB9 was less than 4%. Additionally, amplification appeared to be the primary genetic alteration type in the majority of cancers. Notably, in cholangiocarcinoma, ocular melanoma, pancreatic cancer, and glioma, amplification was the sole observed genetic alteration of PSMB9. These findings highlight the distinct patterns of PSMB9 genetic alterations across different tumor types, with a marked predominance of deep deletion in mature B-cell neoplasms contrasting with the prevalence of amplification in most other malignancies. Beyond genetic alterations, epigenetic regulatory mechanisms, particularly RNA modifications, are crucial for modulating gene expression. We therefore analyzed 44 RNA modification-associated genes across three categories (m1A, m5C, and m6A) in various tumors (Figure 3B). The results demonstrated that PSMB9 exhibited a significant positive correlation with genes involved in these three epigenetic modifications in ACC, PAAD, UVM, KICH, KIRC, KIPAN, COAD, COADREAD, LIHC, and OV. Conversely, in ALL, THYM, WT, CESC, LUSC, and THCA, there was a trend toward negative correlation, primarily associated with m1A and m6A modifications. Though with no experimental validation of RNA modification status or direct molecular interactions, these transcriptional correlations may imply that PSMB9 could have complex, context-dependent associations with RNA epigenetic modification features across tumors. Given the above results, we further explored its correlation with three representative tumor stemness indices (DNAss, EREG-METHss, and ENHss) (Figure 3C–E). The analysis revealed that, in the majority of tumors, PSMB9 expression was significantly negatively correlated with tumor stemness. This observation implies a potential role of PSMB9 in regulating tumor stemness during tumor development and progression.

### 3.4. PSMB9 Associates with Tumor Immune Responses

**Figure 4:**
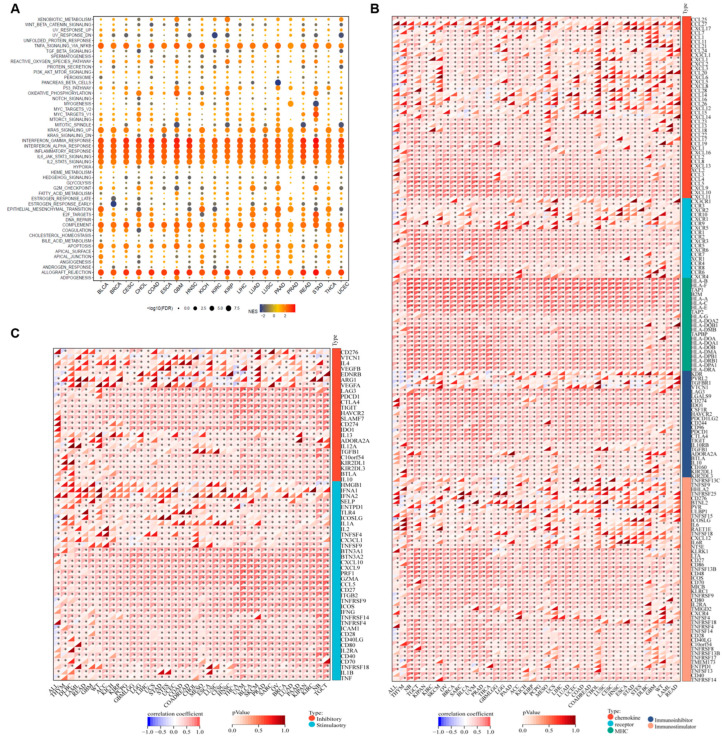
PSMB9 modulates immune-related processes: (**A**) Cancer hallmark gene sets associated with PSMB9 identified by gene set enrichment analysis (GSEA). The normalized enrichment score (NES) for each enriched term is indicated by color intensity, and the false discovery rate (FDR) value is represented by the size of each dot. (**B**) Correlation between PSMB9 expression and immune regulatory genes, including chemokines, immune receptors, MHC, immunoinhibitors, and immunostimulators. (**C**) Pearson correlation analysis between PSMB9 and immune checkpoints. * *p* < 0.05.

To explore the biological processes correlated with PSMB9 expression, we defined the top and the bottom 30% PSMB9 expression subgroups in each cancer type as high and low-PSMB9 subgroups, respectively, and performed a differential expression analysis between the high and low-PSMB9 subgroups in each cancer type with the differential expression genes (DEGs) located (Supplemental Table S2). Based on the DEGs, we performed GSEA across all cancer types to investigate cancer hallmarks associated with PSMB9 (Figure 4A). Immune-related pathways including TNFA-signaling-via-NFKB, IFN-γ response, IFN-α response, inflammatory response, IL6-JAK-STAT signaling, IL2-STAT5 signaling, and complement and allograft-rejection pathways are significantly enriched during high PSMB9 expression in almost all cancer types analyzed, suggesting that PSMB9 could be correlated to interactions between tumor and immune cells. We also found that PSMB9 was associated with KRAS signaling (up) and p53 pathways, which indicates that PSMB9 could be related to tumor genesis. We also notice that, in BLCA, HNSC, LAML, LIHC, OV, PAAD, PCPG, TGCT, and UVM, the abnormal expression of PSMB9 seems to be involved in epithelial–mesenchymal transformation, which plays a critical role in tumor invasion. These findings indicate that PSMB9 could exert a significant role in anti-tumor immune processes.

To further investigate the association between PSMB9 and tumor immunity, we performed Pearson correlation analyses to assess the relationship between PSMB9 and a total of 150 immune pathway regulatory genes across various tumor types using data from the UCSC Xena database and found that PSMB9 was closely correlated with five categories of immune pathway regulatory genes—including chemokines, receptors, MHC molecules, immunoinhibitors, and immunostimulators—in nearly all tumor types (Figure 4B). This widespread correlation suggests that PSMB9 may exert a significant role in antigen presentation, cytokine/chemokine signaling cascades, and immune checkpoint pathways during anti-tumor immune responses.

Building on the above immune-pathway associations, we extended our analysis to explore the correlation between PSMB9 and 60 immune checkpoint-related genes, including 24 inhibitory and 36 stimulatory checkpoint genes (Figure 4C). PSMB9 displayed strong correlations with classical inhibitory molecules such as LAG3, PDCD1, CTLA4, TIGIT, HAVCR2, SLAMF7, and PD-L1 across nearly all tumor types. For the stimulatory immune checkpoints, PSMB9 was similarly strongly associated with most stimulatory checkpoints involved in anti-tumor functions, including IL2, TNFSF4, GZMA, CD27, CCL5, IFNG, ICAM1, CD28, and CD40LG, across nearly all tumor types. Collectively, these results provided evidence that PSMB9 could play a pivotal role in cancer immune responses, prompting us to further investigate its function within the tumor microenvironment (TME).

### 3.5. Higher PSMB9 Reveals Superior Tumor Immune Cell Infiltration

**Figure 5:**
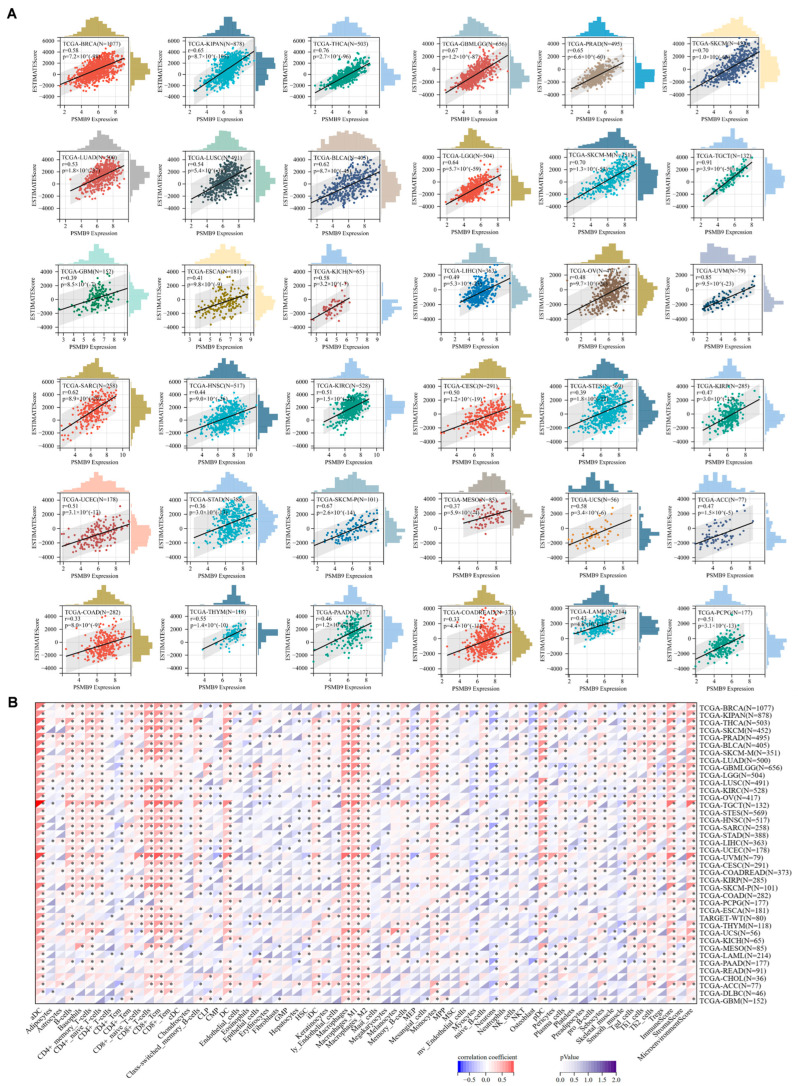
PSMB9 expression correlates with immune cell infiltration in cancers: (**A**) Correlation analysis of PSMB9 expression and immune infiltration score in 36 different tumor types. (**B**) Pearson’s correlation analysis between PSMB9 expression and immune infiltration levels of multiple immune cells in all TCGA cancers. * *p* < 0.05.

As PSMB9 could be closely related to immune responses, interfering with cancer occurrence and progression, we then investigate the correlation between PSMB9 expression level and immune cell infiltration. Significantly positive correlation between PSMB9 expression and immune cell infiltration score was observed in 36 tumor types (Figure 5A). We also downloaded TCGA pan-cancer project data, which employed information from multiple quantitative immune cell infiltration platforms. Our results revealed that PSMB9 was positively correlated with several kinds of immune cells, particularly CD8+ T cells, including CD8+ naïve T cells, effector memory CD8+ T cells, and central memory CD8+ T cells. PSMB9 expression also showed a consistently positive relation to dendric cells and macrophages in most tumor types (Figure 5B). Due to these findings, we reasonably inferred that PSMB9 could participate in responses to immunotherapies, especially those based on T cells.

### 3.6. PSMB9 Modulates Responses to ICIs

**Figure 6:**
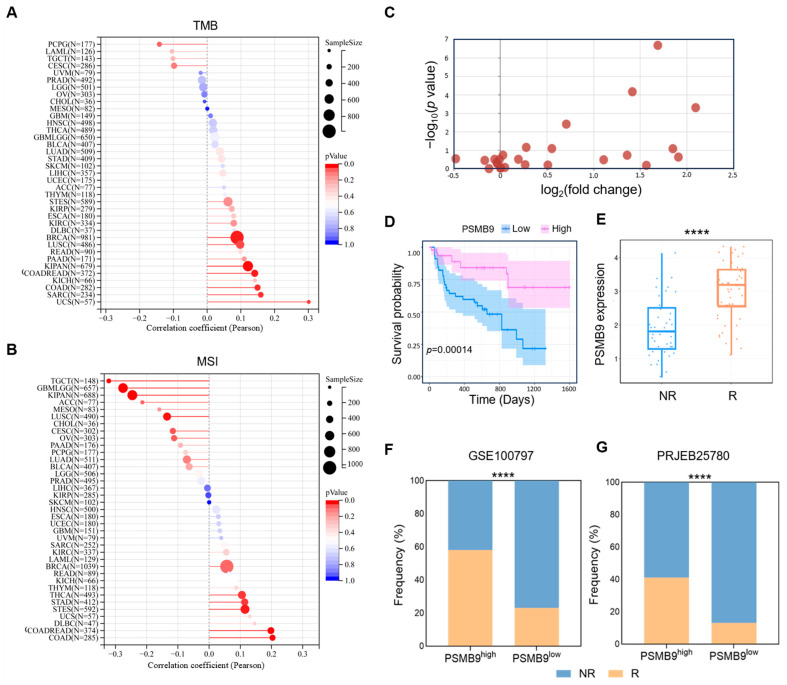
PSMB9 correlates with immunotherapy response: (**A**) Pearson correlation between PSMB9 expression and TMB. (**B**) Pearson correlation between PSMB9 expression and MSI. TMB, tumor mutation burden; MSI, microsatellite instability. The size of the circles corresponds to sample sizes. The color gradient of the bars indicates the significance of the *p* value, with the red color corresponding to a more significant *p* value. (**C**) Differential PSMB9 expression between responders and non-responders from 25 immunotherapy cohorts, with details of cohorts described in Supplementary Table S3. A Log_2_(fold change) > 0 indicates that PSMB9 expression in the R group is higher than that in the NR group (upregulation), with a larger positive value representing a more significant upregulation magnitude. A higher −log_10_(*p* value) corresponds to a smaller *p* value and stronger statistical significance. (**D**) Kaplan–Meier curve of low and high-PSMB9 subgroups in melanoma patients taking anti-PD-1 and anti-CTLA-4 plus anti-PD-1treatment (PRJEB23709). (**E**) Differential PSMB9 expression in responders (patients who achieved complete response or partial response) and non-responders (patients who achieved stable disease or progressive disease) from cohort PRJEB23709. (**F**,**G**) The proportion of responders and non-responders in the low-PSMB9 and high-PSMB9 subgroups in melanoma patients taking ACT treatment (GSE100797) and stomach adenocarcinoma patients taking anti-PD-1 treatment (PRJEB25780). R, responders; NR, non-responders. **** *p* < 0.0001.

Based on the role of PSMB9 in immune cell infiltrations as well as immune-activated pathways and biological processes, we hypothesized that PSMB9 might play a prognostic role in immunotherapy responses. Tumor mutational burden (TMB) and microsatellite instability (MSI) are established critical biomarkers for ICI response [31,32]; thus, we further explored the correlation between PSMB9 expression and these genomic features across multiple cancer types. Our analyses revealed that PSMB9 expression was significantly positively correlated with TMB in the majority of tumor types, with particularly strong associations observed in USC, SARC, COAD, COADREAD, KIPAN, and BRCA (Figure 6A). In contrast, the correlation between PSMB9 and MSI displayed distinct tumor-specific patterns. Positive correlations were observed in COAD, COADREAD, STES, STAD, and THCA, while negative correlations were noted in TGCT, GBMLGG, KIPAN, and LUSC (Figure 6B).

Our findings of PSMB9 in immune infiltrations indicated a T cell-dominated enrichment in the TME. We speculated that PSMB9 could be particularly predictive in T cell-based immunotherapies. To address this hypothesis, we retrieved treatment regimens and clinical outcome data from 25 independent immunotherapy cohorts within the TIGER database, followed by a systematic analysis of differential PSMB9 expression profiles between patients who responded to treatment and those who did not. Across these diverse cohorts encompassing various cancer types and therapeutic strategies, PSMB9 expression was consistently upregulated in responders relative to non-responders, with collective elevation of its expression levels in treatment-responsive groups, thereby providing initial evidence for a potential association with treatment response (Figure 6C, Supplementary Table S3). To validate this observation, we selected the representative cohort of melanoma patients treated with anti-PD-1, anti-PD-L1 plus anti-CTLA-4 treatment (PRJEB23709) [26]. Patients were stratified into high- and low-PSMB9 expression subgroups based on the median expression level of PSMB9, and survival curves for these subgroups were generated. Kaplan–Meier analysis revealed significantly improved survival in the PSMB9^high^ compared to the PSMB9^low^ subgroup (Figure 6D). We further stratified that PSMB9 expression levels were significantly higher in the treatment-responsive group compared with the non-responsive group, reinforcing the association between PSMB9 upregulation and favorable therapeutic outcomes.

To generalize these findings across different tumor types and immunotherapeutic strategies, we then calculated the proportion of responders and non-responders in high- and low-PSMB9 subgroups within melanoma patients receiving adoptive cell transfer (ACT) therapy (GSE100797) [33] and STAD patients treated with anti-PD-1 therapy (PRJEB25780) [25] (Figure 6F,G). In both cohorts, the proportion of patients responding to immunotherapy was significantly higher in the PSMB9^high^ subgroup compared to the PSMB9^low^ subgroup. These results collectively highlight the potential of PSMB9 as a predictive indicator of immunotherapeutic efficacy across diverse tumor types and treatment approaches.

To compare the predictive performance of PSMB9 with previously reported immune response signatures [34,35], we analyzed the anti-PD-1 therapy data from a melanoma and a STAD cohort (PRJEB23709 and PRJEB25780) [25,26]. The performance of PSMB9 in the melanoma cohort was comparable to that of previously reported signatures such as T cell-inflamed GEP, TAM M2, IFNG, CD8, and CD274, which all showed an AUC more than 0.8. In the STAD cohort, PSMB9 showed a predictive performance second only to CD8 (Table 1). Taken together, these findings underscore a multifaceted role of PSMB9 in modulating responses to immunotherapy, with its expression levels exhibiting predictive value across diverse tumor types and therapeutic approaches.

### 3.7. PSMB9 Plays a Potential Role in CAR-T Cell Therapy

**Figure 7:**
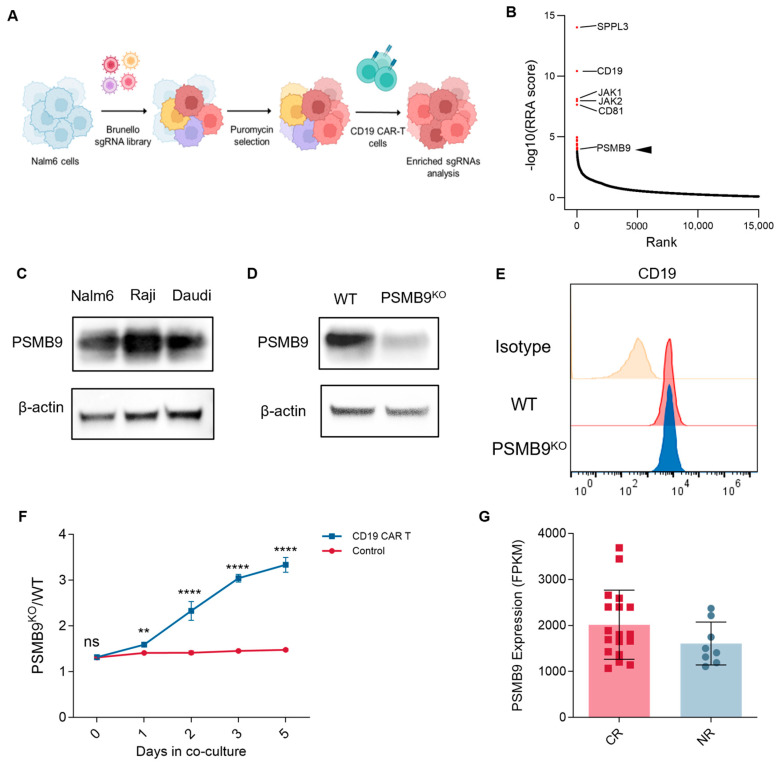
PSMB9 loss in tumor cells negatively impacts CAR T cell efficacy: (**A**) Schema of genome-wide CRISPR/Cas9 screening. (**B**) Genes of the most significant sgRNA enrichment. (**C**) Western blot analysis of PSMB9 protein levels in CD19^+^ B-lymphoid cell lines. (**D**) Western blot validation of PSMB9 knockout in Nalm6 cells. (**E**) Flow cytometry analyzes CD19 expression in WT and PSMB9^KO^ Nalm6 cells. (**F**) PSMB9^KO^ /WT Nalm6 cell ratio in the growth competitive assay. PSMB9^KO^ Nalm6 cells (labeled in GFP) and WT Nalm6 cells (labeled in mCherry) are mixed at an approximately 1:1 ratio, with CD19 CAR-T cells added to the experimental group at an E:T ratio of 1:8 (*n* = 3). The PSMB9^KO^ (GFP^+^)/ WT (mCherry^+^) ratio is evaluated by flow cytometry over time. Three independent experiments are performed, and representative results are shown. Statistical comparisons are made via a two-way ANOVA test with multiple comparisons. Data are presented as mean ± standard deviation. ** *p* < 0.01; **** *p* < 0.0001. ns indicates not significant (*p* > 0.05). (**G**) PSMB9 expression levels in the leukemia-infiltrated bone marrow samples from patients who achieved complete remissions and who did not respond to CAR-T cell therapy (GSE153670). WT, wild type. RRA, robust rank aggregation.

CAR-T cell therapy has revolutionized cancer treatment, particularly for hematological malignancies, by harnessing genetically engineered T cells to recognize and eliminate tumor cells expressing specific antigens [36,37]. To this day, tumor resistance to CAR-T cell therapy remains a major factor leading to patient recurrence, and identifying effective predictive molecules for CAR-T cell therapy resistance is crucial. Given PSMB9′s established roles in modulating T cell infiltration, regulating immune response pathways, and influencing immune checkpoint molecule expression, we hypothesized that PSMB9 might also impact the efficacy of CAR-T cell therapy, extending beyond its previously identified associations with ICIs. To systematically identify genetic determinants of resistance to CAR-T cell therapy, we have performed an unbiased genome-scale CRISPR/Cas9 screening in Nalm6 cells cocultured with CD19 CAR-T cells, aimed to identify pivotal regulators that determined resistance to CAR-T cell therapy (Figure 7A). In our screening, CAR-T cells were supplemented to Nalm6 cells every 3 days at a very low effector: target ratio of 1:50 to simulate the actual high-burden in vivo environment. After deep sequencing and analysis by the MAGeCK algorithm, we identified that PSMB9 was among the top 20 most significantly enriched sgRNAs (Figure 7B). To investigate the impact of tumor-intrinsic PSMB9 expression on CD19 CAR-T cell therapy, we first examined PSMB9 protein levels across CD19^+^ ALL cell line Nalm6 and lymphoma cell lines Raji and Daudi using Western blot analysis. Leveraging the CRISPR/Cas9 system, we generated PSMB9 knockout (PSMB9^KO^) Nalm6 cells and confirmed ablation of PSMB9 expression at the protein level (Figure 7D). Seven days post-knockout, flow cytometric analysis revealed no significant alteration in CD19 surface expression in PSMB9^KO^ Nalm6 cells, indicating that PSMB9 ablation did not mediate changes in the target antigen. To directly assess the functional consequences of PSMB9 loss on CAR-T cell efficacy, we performed a competitive coculture assay (Figure 7F). mCherry-labeled control Nalm6 cells and GFP-labeled PSMB9^KO^ Nalm6 cells were cocultured with CD19 CAR-T cells at an E:T ratio of 1:8, and the ratio of PSMB9^KO^ to control Nalm6 cells was monitored over time using flow cytometry. Notably, in the presence of CD19 CAR-T cells, the proportion of PSMB9^KO^ Nalm6 cells progressively increased relative to control cells, suggesting that PSMB9 deficiency could confer a survival advantage under CAR-T cell pressure.

To further investigate the association between PSMB9 expression levels and clinical outcomes of CAR-T cell therapy, we evaluated the bulk RNA sequencing data of leukemia-infiltrated bone marrow samples collected prior to CD19 CAR-T therapy from a previously reported study [38]. Although not statically significant, the expression level of PSMB9 in patients who achieved complete remissions tended to be higher than in patients who did not respond to CAR-T cell therapy, suggesting a potential role of PSMB9 in CAR-T cell therapy outcome. Taken together, PSMB9 could be associated with the sensitivity of tumor cells to CAR-T cell therapy, suggesting that the loss of PSMB9 might be a novel molecular mechanism for tumor cells to escape from CAR-T cells.

## 4. Discussion

Despite the remarkable progress in cancer immunotherapy, particularly with ICIs and adoptive cell therapies, the clinical efficacy remains limited by inadequate biomarkers for patient stratification and a lack of mechanistic understanding of tumor-immune crosstalk [39]. Current biomarkers such as PD-L1 expression and TMB often fail to capture the full complexity of the tumor immune microenvironment [40,41,42], leading to inconsistent responses across cancer types. While proteasome components have been implicated in immune surveillance, the role of PSMB9, the key subunit of the immunoproteasome, has been studied primarily in specific contexts like NSCLC, with limited systematic analysis of its pan-cancer expression, role in orchestrating tumor immune environment, and functional relevance to immune-mediated therapies. In this study, we systematically characterized PSMB9 across human cancers, uncovering its dysregulated expression, prognostic significance, and critical involvement in shaping the tumor immune landscape.

Our analyses revealed that PSMB9 is aberrantly expressed in most malignancies, with context-dependent associations with tumor stage and patient survival. Genetically, PSMB9 alterations vary by cancer type, with amplification predominating in most tumors and deep deletions in mature B-cell neoplasms, while epigenetically, it correlates with RNA modification pathways, suggesting layered regulatory mechanisms. Functionally, PSMB9 is tightly linked to immune-related pathways, including IFN-γ responses and antigen presentation, and its expression positively correlates with infiltration of CD8+ T cells, dendritic cells, and macrophages in the TME. Importantly, PSMB9 predicts response to ICIs across diverse cohorts and modulates sensitivity to CAR-T cell therapy, emerging as a promising biomarker for T cell-based immunotherapies.

The efficacy of antitumor immunotherapy is largely dependent on responses of tumor-specific immune cells [43]. Our report suggests that PSMB9 could play a crucial role in tumor immunogenicity and immunotherapy responsiveness across a variety of cancer types. GSEA results showed significantly upregulated immune-related pathways, aligned with the role of PSMB9 as a critical immunoproteasome subunit that assists in generating the peptide epitopes for MHC-I presentation [44]. Increased immunogenic antigens resulting from higher PSMB9 expression could explain the significantly enriched tumor-infiltrated immune cells, represented by CD8^+^ T cells, which play a central role in antitumor activity in the TME. Enrichment of tumor-infiltrated T cells accompanied by higher antigen representation levels could cause increased chemokine and cytokine responses and lytic secretions, in turn leading to superior tumor cytotoxicity. Suppressed MHC-I antigen processing and presentation machinery allowed cancer cells to evade CD8^+^ T cell-mediated immunosurveillance, which could explain resistance to immunotherapy in PSMB9 low tumors [1,45].

Furthermore, our findings regarding the predictive value of PSMB9 in predicting the response to immunotherapy are significant. We found that higher PSMB9 expression tends to be associated with improved survival outcomes in patients who received ICIs, possibly due to enhanced antigen presentation and subsequent immunogenic cell death. This correlation could stem from the heightened immune system activity against tumors with high mutational burden, facilitating heightened antigenicity and immune clearance. Also, PSMB9 showed comparable predictive performance compared with known immune response signatures like CAF, TLS, IFNG, etc. Our analysis suggests that PSMB9 expression might be considered a useful biomarker for selecting patients who are likely to benefit from ICIs. Furthermore, CRISPR/Cas9 screening revealed that PSMB9 was among the top 20 most significantly enriched sgRNA. The competitive growth assay revealed that PSMB9 deficiency confers resistance to CAR-T cell therapy, likely by impairing antigen processing or T cell activation. This is the first report of the predictive role of PSMB9 in CAR-T therapy, suggesting that the lack of PSMB9 in tumor cells could possibly lead to acquired CAR-T cell resistance. Further experiments focusing on how PSMB9 interferes with CAR-T cell phenotype and functions are needed. Together, these findings position PSMB9 as a unifying biomarker for both ICIs and CAR-T therapies, addressing a critical need for tools to identify patients most likely to benefit from T cell-mediated interventions. However, in individual tumors where PSMB9 exhibits a negative correlation with prognosis, it conversely shows a positive correlation with the efficacy of immunotherapy. Tumor prognosis could be influenced by a variety of factors, and this seemingly paradoxical result highlights the complexity of the tumor immune microenvironment and the regulatory functions of PSMB9.

Another aspect of PSMB9 biology uncovered in this study is its complex genetic and epigenetic regulation, coupled with its inverse correlation with tumor stemness, which sheds light on its role in tumorigenesis beyond immune modulation. Genetic alteration analysis revealed that PSMB9 is predominantly amplified in most cancers, while mature B-cell neoplasms exhibit a high rate of deep deletions, suggesting distinct selective pressures across tumor types. This dichotomy may reflect context-specific dependencies: amplification could enhance PSMB9-mediated antigen presentation in solid tumors, whereas deletion in hematological malignancies might disable immune recognition. Epigenetically, PSMB9′s correlation with m1A, m5C, and m6A RNA modification genes further indicates layered regulatory control, with positive associations in cancers like PAAD and KIRC potentially facilitating immune-related mRNA stability, while negative correlations in THCA and CESC might dampen its expression to promote immune evasion. Notably, the consistent negative correlation between PSMB9 expression and tumor stemness indices across most tumors suggests a previously unrecognized role in suppressing stem cell-like properties. Cancer stem cells are critical for tumor initiation, metastasis, and therapy resistance, and their elimination is a key goal of effective cancer treatment [46]. PSMB9′s inverse association with stemness could arise from enhanced immune clearance of stem-like cells, as PSMB9-dependent antigen presentation would make these cells more visible to cytotoxic T cells. Alternatively, PSMB9 might directly regulate stemness pathways, though further studies are needed to dissect this relationship. This link between PSMB9, immune competence, and stemness provides a mechanistic basis for its prognostic value, as tumors with lower stemness and higher PSMB9 expression are likely more susceptible to immune-mediated destruction and conventional therapies.

However, several limitations must be noted in our study, including potential biases introduced by the retrospective nature of data collection across diverse cohorts with varying treatment regimens and follow-up periods. For different cohorts, differences in cancer types, disease stages, and treatment regimens (monotherapy vs. combination therapy, first-line vs. subsequent lines) could bring treatment heterogeneity across diverse patient cohorts, which may influence the observed associations. Moreover, how PSMB9 influences the immune landscape within various tumor types remains insufficiently elucidated and warrants further molecular and immunological investigations. In our subsequent research, we will also conduct further investigations into the specific molecular mechanisms by which tumor PSMB9 deficiency interferes with CAR T cell efficacy.

Generally, our study provided a comprehensive pan-cancer analysis that underscores the potential of PSMB9 as a valuable biomarker for cancer prognosis and prediction of T cell-based immunotherapy response. Further validation and investigation into its molecular mechanisms are imperative, especially its interaction with other immune components within the TME, to enhance the understanding of the role of PSMB9 across various cancer types and under different therapeutic conditions to ensure effective, personalized treatment strategies.

## 5. Conclusions

Our findings identify PSMB9 as a key immune modulator with prognostic roles especially in T cell-based immunotherapies. PSMB9 shows widespread dysregulation, predominantly upregulated in most malignancies and linked to specific pathological stages, correlating with divergent survival outcomes across cancers. Mechanistically, it markedly activates immune-related pathways (e.g., IFN response, JAK-STAT signaling, IL2-STAT5 signaling), enhances T cell infiltration, and also correlates with tumor stemness. Critically, PSMB9 predicts superior response to ICIs and could be potentially associated with CAR-T sensitivity, with its deficiency driving resistance. Functional validation confirms its role in CAR-T efficacy. Despite limitations in cohort heterogeneity, this study positions PSMB9 as a pivotal orchestrator of tumor immune landscapes, highlighting its potential as a biomarker and therapeutic target to advance precision immunotherapy.

## Figures and Tables

**Table 1:** Performance comparison with known immune response signatures.

Signature Name	Description	AUC (Melanoma)	AUC (STAD)
PSMB9	PSMB9	0.8164	0.7143
T cell-inflamed GEP	CCL5, CD27, CD274, CD276, CD8A, CMKLR1, CXCL9, CXCR6, HLA-DQA1, HLA-DRB1, HLA-E, IDO1, LAG3, NKG7, PDCD1LG2, PSMB10, STAT1	0.8229	0.7043
CAF	Cancer-associated fibroblasts	0.5829	0.5731
TAM M2	Tumor-associated macrophages	0.8229	0.6174
IFNG	CXCL10, CXCL9, HLA-DRA, IDO1, IFNG, STAT1	0.818	0.6901
CD8	CD8A, CD8B	0.8438	0.7519
CD274	CD274	0.8647	0.6817
TLS	CCL19, CCL21, CXCL13, CCR7, SELL, LAMP3, CXCR4, CD86, BCL6	0.7327	0.6383
TLS-melanoma	CD79B, CD1D, CCR6, LAT, SKAP1, CETP, EIF1AY, RBP5, PTGDS	0.7279	0.4662
T cell dysfunction	Genes regulating dysfunction of T cells in TME	0.6844	0.5656
T cell exclusion	Genes regulating T cell exclusion in TME	0.7746	0.5622
MDSC	Myeloid-derived suppressor cells	0.6924	0.4353

## Data Availability

The datasets utilized in this study can be accessed from the following sources: The Cancer Genome Atlas (https://cancergenome.nih.gov/, accessed on 6 June 2025), GTEx (https://www.gtexportal.org/, accessed on 6 June 2025), cBioPortal (http://www.cbioportal.org, accessed on 10 June 2025), GEPIA2 (http://gepia2.cancer-pku.cn/, accessed on 30 June 2025), UCSC Xena database (https://xenabrowser.net/, accessed on 6 June 2025), Tumor Immunotherapy Gene Expression Resource (TIGER) database (http://tiger.canceromics.org/, accessed on 16 June 2025), and the Human Protein Atlas (https://www.proteinatlas.org/, accessed on 16 June 2025).

## Supplementary Materials

The following supporting information can be downloaded at https://www.mdpi.com/article/10.3390/cimb47090712/s1.

## Abbreviations

The following abbreviations are used in this manuscript:

PSMB9	Proteasome subunit beta type-9
MHC	Major histocompatibility complex
CAR T	Chimeric antigen receptor T
NSCLC	Non-small-cell lung cancer
GTEx	Genotype-tissue expression
IHC	Immunohistochemical
HPA	Human Protein Atlas
TCGA	The Cancer Genome Atlas
GBM	Glioblastoma multiforme
GBMLGG	Glioma (including GBM and LGG)
LGG	Brain lower-grade glioma
BRCA	Breast invasive carcinoma
CESC	Endocervical adenocarcinoma
LUAD	Lung adenocarcinoma
ESCA	Esophageal carcinoma
STES	Stomach and esophageal carcinoma
KIRP	Kidney renal papillary cell carcinoma
COAD	Colon adenocarcinoma
PRAD	Prostate adenocarcinoma
STAD	Stomach adenocarcinoma
HNSC	Head and neck squamous cell carcinoma
KIRC	Kidney renal clear cell carcinoma
LIHC	Liver hepatocellular carcinoma
SKCM	Skin cutaneous melanoma
BLCA	Bladder urothelial carcinoma
THCA	Thyroid carcinoma
READ	Rectum adenocarcinoma
OV	Ovarian serous cystadenocarcinoma
PAAD	Pancreatic adenocarcinoma
TGCT	Testicular germ cell tumors
LAML	Acute myeloid leukemia
KICH	Kidney chromophobe
CHOL	Cholangiocarcinoma
UCS	Uterine carcinosarcoma
ALL	Acute lymphoblastic leukemia
WT	Wilms tumor
OS	Overall survival
UVM	Uveal melanoma
KIPAN	Pan-kidney cancer cohort (including KIRC, KIRP, KICH)
SKCM-M	Skin cutaneous melanoma-metastatic
SARC	Sarcoma
DSS	Disease-specific survival
DFI	Disease-free interval
PFI	Progression-free interval
THYM	Thymoma
SKCM-P	Skin cutaneous melanoma-primary
DNAss	DNA stemness score
EREG-METHss	Epigenetic regulation of stemness-methylation score
ENHss	Enhancer stemness score
DEGs	Differential expression genes
GSEA	Gene set enrichment analysis
NFKB	Nuclear factor kappa-B
TMB	Tumor mutational burden
MSI	Microsatellite instability
ICIs	Immune checkpoint inhibitors
TIGER	Tumor Immunotherapy Gene Expression Resource
ACT	Adoptive cell transfer
SNV	Simple nucleotide variation
CRISPR	Clustered regularly interspaced short palindromic repeat
sgRNA	Single-guide RNA
KO	Knockout
PBMCs	Peripheral blood mononuclear cells
ECL	Enhanced chemiluminescence
