# Supplementary material for: PSMB9 Orchestrates Tumor Immune Landscape and Serves as a Potent Biomarker for Prognosis and T Cell-Based Immunotherapy Response
Source: Curr Issues Mol Biol. 2025 Sep 1;47(9):712. doi: 10.3390/cimb47090712 (PMC12468175; doi:10.3390/cimb47090712)
Supplement: Supplementary file 1 [file cimb-47-00712-s001.zip › Supplementary Materials Figures S1-S3.pdf]

Supplemental Figure S1

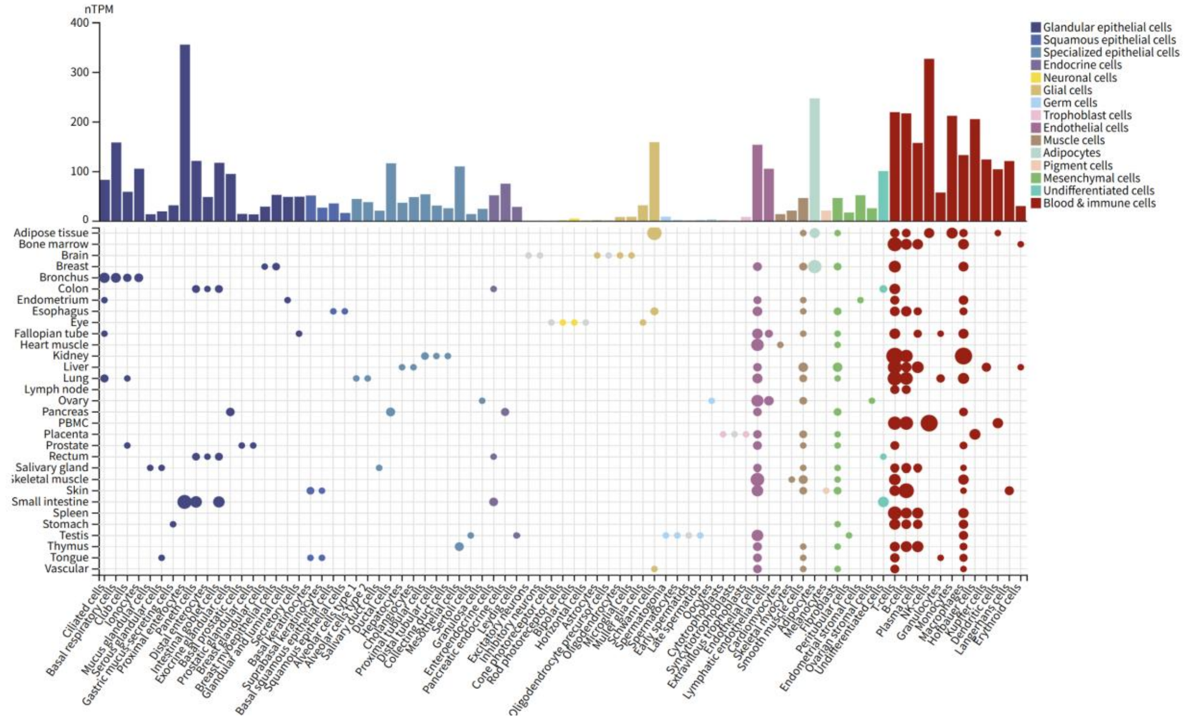

Supplemental Figure S1. Normalized single cell RNA expression (nTPM) across diverse normal tissues. Cell type groups are color-coded, each comprising cell types sharing common functional characteristics.

Supplemental Figure S2

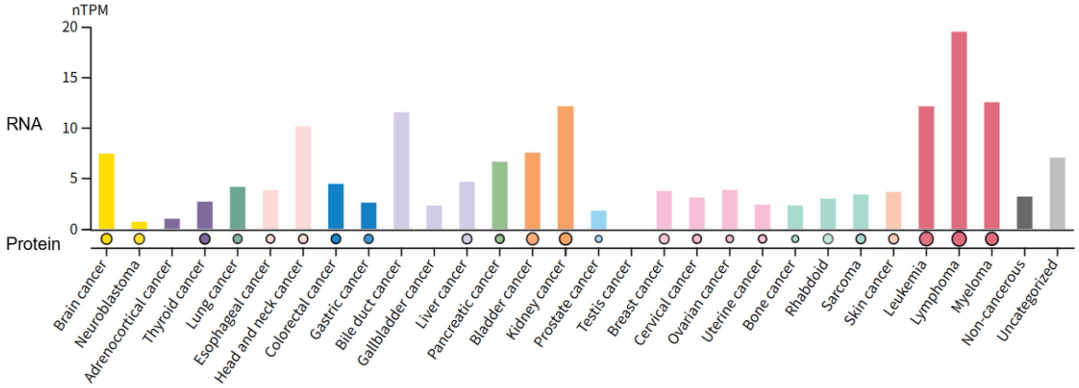

Supplemental Figure S2. RNA level and protein level of various cell line groups of PSMB9. RNA expression data was shown as normalized transcript per million (nTPM) values of cancer cell lines, which were grouped according to cancer type. Protein expression across cancer cell lines derived from mass spectrometry (MS) proteomics data of the HPA dataset is visualized as circles for each cancer cell line group, with circle size denoting the median Normalized Relative Protein Expression (nRPX) of cell lines, white circles indicating proteins undetected by MS in corresponding cell lines, and absence of a circle signifying missing MS data for the cell line group.

Supplemental Figure S3

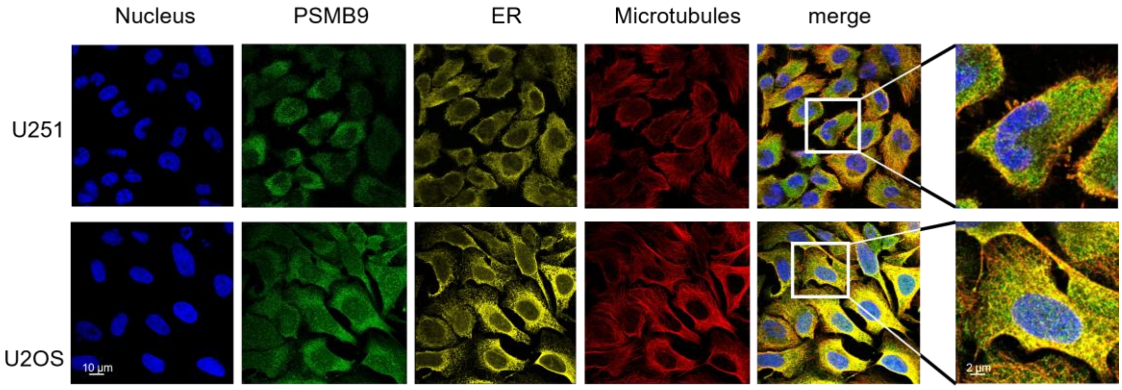

Supplemental Figure S3. The intracellular localization of PSMB9. Representative immunofluorescence images showing the subcellular localization of PSMB9 (green) in U251 and U2OS cell lines, co-stained with markers for the nucleus (blue), endoplasmic reticulum (yellow), and microtubules (red).
